# Comparative Genomics Analysis of Rice and Pineapple Contributes to Understand the Chromosome Number Reduction and Genomic Changes in Grasses

**DOI:** 10.3389/fgene.2016.00174

**Published:** 2016-10-04

**Authors:** Jinpeng Wang, Jiaxiang Yu, Pengchuan Sun, Yuxian Li, Ruiyan Xia, Yinzhe Liu, Xuelian Ma, Jigao Yu, Nanshan Yang, Tianyu Lei, Zhenyi Wang, Li Wang, Weina Ge, Xiaoming Song, Xiaojian Liu, Sangrong Sun, Tao Liu, Dianchuan Jin, Yuxin Pan, Xiyin Wang

**Affiliations:** ^1^Center for Genomics and Computational Biology, School of Life Sciences, North China University of Science and TechnologyTangshan, China; ^2^College of Science, North China University of Science and TechnologyTangshan, China

**Keywords:** rice, pineapple, grass, chromosome, genome

## Abstract

Rice is one of the most researched model plant, and has a genome structure most resembling that of the grass common ancestor after a grass common tetraploidization ∼100 million years ago. There has been a standing controversy whether there had been five or seven basic chromosomes, before the tetraploidization, which were tackled but could not be well solved for the lacking of a sequenced and assembled outgroup plant to have a conservative genome structure. Recently, the availability of pineapple genome, which has not been subjected to the grass-common tetraploidization, provides a precious opportunity to solve the above controversy and to research into genome changes of rice and other grasses. Here, we performed a comparative genomics analysis of pineapple and rice, and found solid evidence that grass-common ancestor had 2n = 2x = 14 basic chromosomes before the tetraploidization and duplicated to 2n = 4x = 28 after the event. Moreover, we proposed that enormous gene missing from duplicated regions in rice should be explained by an allotetraploid produced by prominently divergent parental lines, rather than gene losses after their divergence. This means that genome fractionation might have occurred before the formation of the allotetraploid grass ancestor.

## Introduction

Genetic integrity is well preserved majorly through condense packing genetic materials into chromosomes (Ding et al., 2004; McKee, 2004; Jordan, 2006). In spite of few variations, the numbers of chromosomes in eukaryotes often varies in a relatively small range. In grass family, though some plants have 100 of chromosomes, e.g., in cultivated sugarcane, the basic chromosome numbers lay in a range from *n* = 2 to 17 (Grass-phylogeny-working-group-II, 2012). For example, rice. Sorghum, maize, and wheat have *n* = 12, 10, 10, and 7 basic chromosomes, respectively. A small chromosome number may help maintain the efficiency of homologous chromosome pairing and segregating, avoiding likely mispairing and twisting (Vazquez et al., 2002; Nicolas et al., 2008).

As to the ancestral chromosome number, it was proposed that the grass common ancestor might have seven basic chromosomes, or 2n = 2x = 14 chromosomes before a grass-common tetraploidization (Wang et al., 2016). After the genome-doubling events, and following wide-spread chromosomal rearrangement, the number of basic chromosomes reduced to 12, a number preserved in rice but further reduced in many other grasses, e.g., sorghum, barley, wheat, and *Brachypodium*. This phenomenon occurred in maize, too, though it was affected by another whole-genome doubling specific to itself. To explain how chromosome number was reduced in grasses and other plants, even all eukaryotes, a novel genome repatterning theory was proposed, emphasizing the importance of removal of telomeres through intra- or inter-chromosomal DNA crossing-over (Wang et al., 2016). It showed the mechanism how chromosome fusion might have occurred, and stated that a fusion of two chromosomes would be accompanied by the production of a satellite chromosome, formed by two telomeres from the same chromosome or from two different chromosomes. Based on this fusion theory, the evolutionary trajectories of chromosomes were reconstructed along the main lineages of grasses.

However, there was another proposition that there might be five basic chromosomes before the grass-common genome doubling (Murat et al., 2010). This proposition, or a fission theory, was based on the inference that some ancestral chromosomes might have been split to produce smaller chromosomes. In contract, the above fusion model predicted that several ancestral chromosomes might be merged to produce larger chromosomes.

A comparative analysis of rice genome and the monocot relative, banana, genome was performed, to find the likelihood of independent entities of certain ancestral chromosomes of rice chromosomes 4 and 6, and 7 and 10, in the ancestral genome (Wang et al., 2016). The analysis, to some extent, proved the fusion model, and rejected the fission one. However, due to a complex nature of the banana genome, subjected to three polyploidizations not shared with rice, and far in evolutionary history, banana genome was not so good to provide a consolidate evidence to solve the above controversy. Two polyploidizations in the rice lineage after splitting with banana became further hurdle. Recently, an effort to distinguish two subgenomes, dominant and sensitive ones, let the authors proposing the fission model turned to the hypothesis of seven proto-chromosomes (Murat et al., 2014). However, we still need a solid evidence to solve the controversy.

**FIGURE 1:**
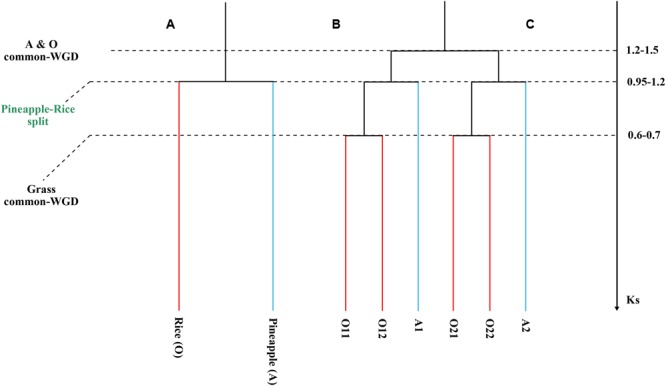
**Phylogenetic tree and gene tree. (A)** A phylogenetic tree with ancestral polyploidizations shown with squares. **(B)** A gene tree to show paralogs in each genome. **(C)** We showed synonymous substitution values for events.

Fortunately, a recent genome sequencing effort deciphered the genome of pineapple (*Ananas comosus*), which has a rather simple relationship with rice (**Figure 1**). After the split of them, rice was subjected by the grass-common genome doubling, while pineapple has not been affected since. This provides precious opportunity to solve the above controversy over grass ancestral chromosome number, and evaluate the effectiveness of the fusion and fission model. Moreover, comparison of them would show divergent genomic changes of them after their split. Furthermore, it will help reconstruct a credible evolutionary history of grasses, a family with several important cereal crops.

## Materials and Methods

### Materials

Genomes and their gene annotations for both plants were downloaded from Joint Genome Institute^1^, and the respective data version of rice is *Oryza sativa v7_JGI*, and pineapple *A. comosus v3.*

### Genomic Homology

To show genome intra- or inter-genomic homology, gene CDSs from one plant were protein-coding searched against its own or another genome sequence using BLASTN. The best, second best, and other matches with *E*-value ≤ 1*e*-5 were displayed in different colors, to help distinguish orthology from paralogy, or layers of paralogy as a result of recursive WGDs. Dot-plots were produced using Perl scripts.

To find gene collinearity, by running BLASTP among genes, we searched for potential anchors (*E*-value ≤ 1*e*-5; top five matches) between every possible pair of chromosomes within rice and pineapple, and between them. By running ColinearScan (Wang et al., 2006), we revealed homologous blocks within each genome and between different genomes (by setting maximal searching gap ≤ 50 genes and *P*-value < 0.05). By characterizing homologous sequence similarities, measured by collinear gene number and sequence identity, we characterized the paralogy and orthology among them.

### Synonymous Nucleotide Substitutions (*K*s)

To calculate the synonymous nucleotide substitution rates (*K*s) between genes, we used the Nei-Gojobori method implemented in PAML package (Yang, 2007) to estimate the values.

## Results

### Inference of Collinear Homologs

By using ColinearScan, we inferred intragenomic homologous genes in collinearity within rice and pineapple, respectively, and intergenomic homologs between them. We counted collinear genes in blocks with different sizes, which was measured by collinear gene numbers in blocks (**Table 1**). In rice, 2243 genes were found in 54 blocks containing more than 10 genes; while in pineapple, there are 1863 collinear genes in 87 blocks. For the large blocks having more than 50 collinear genes, there are 11 and 9 blocks involving 1599 and 555 collinear genes in these two plants, respectively. The largest block in rice was located between chromosomes 2 and 4, contains 268 collinear genes; while the largest block in pineapple was located on chromosomes 1 and 15, containing 79 collinear genes. This shows rice have longer blocks than pineapple. As to colinear gene number, The intergenomic homology between rice and pineapple is much better than intragenomic homology. There are 438 intergenomic blocks, containing 12057 collinear genes, with total 4071 collinear genes from 50 blocks with block size >50 collinear genes. Here, we found that there are much more homologs resided on longer blocks between different genomes. A higher similarity between different genomes makes it valuable to perform intergenomic comparison to understand genome structure of a genome.

**Table 1:** Number of homologous genes and blocks within and between rice and pineapple.

Homologous blocks withinand among genome		Block_lens >4	Block_lens >10	Block_lens >20	Block_lens >50	CGP reside in LDB	LDB on chromosomes
*Oryza sativa*	Block	358	54	17	11	268	OS02–OS04
	Gene	3892	2243	1754	1599		
*Ananas comosus*	Block	363	87	24	9	79	AC01–AC15
	Gene	3363	1863	1038	555		
*A. comosus* vs. *O. sativa*	Block	1517	438	202	50	190	AC06–OS02
	Gene	17956	12057	8865	4071		

*CGP, colinear gene pairs; LDB, longest duplicated block.*

### Classification of Intergenomic Homology

**FIGURE 2:**
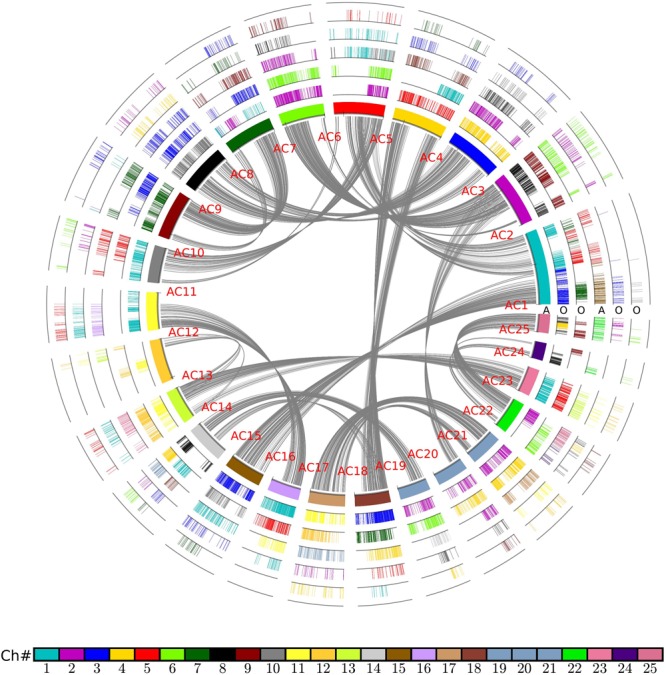
**Homologous alignments of rice genome with pineapple as reference.** Genomic paralogy, orthology, and outparalogy information within and among pineapple (A) and rice (O) are displayed in six circles; The inner circle represents 25 pineapple chromosomes, which are differently colored. A pineapple chromosome block was produced by short lines, each representing a gene, A gene short line is colored as to its source chromosome number in a specific species. A pineapple genomic region has two sets of rice corresponding regions due to grass-common tetraploidy, to form another two circles in sequential order. The shared tetraploidy results in a set of paralogous regions in pineapple, to form another circle to show collinear genes within pineapple genome. This second pineapple circle of regions has its own two sets of rice orthologs, to form another two circles of rice chromosomal regions. The curvy lines in the inner circle show collinear homologs in pineapple genome.

**FIGURE 3:**
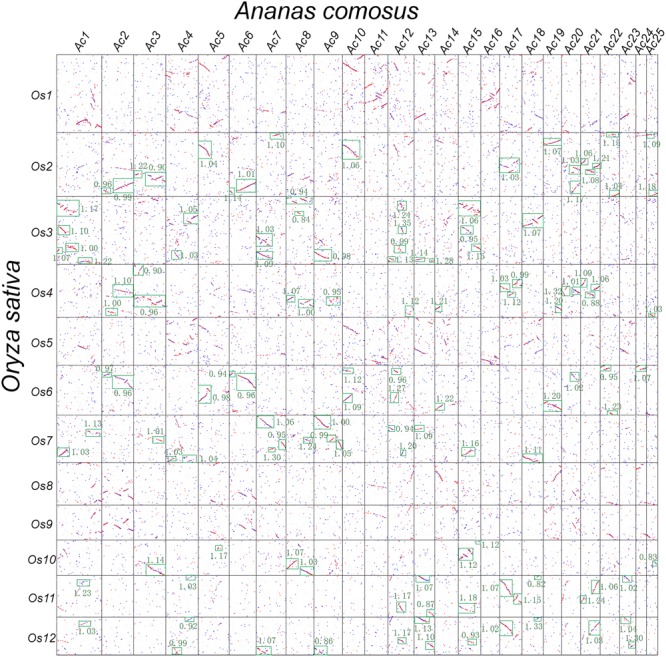
**Dotplot between rice and pineapple.** Rice and pineapple chromosomes are, respectively, aligned horizontally and vertically. Red dots show three homologous pineapple genes best matching a rice gene, and blue dots show other matches. *K*s values are shown in green besides the boxed collinear gene blocks.

As noted above, rice and other grasses share a tetraploidization event after the split with pineapple, which has not been affected by polyploidization ever since. Therefore, there should be 1:2 orthologous gene ratio between pineapple and rice (**Figure 2**). Considering the fact of a more ancient polyploidization, if no gene or DNA losses, we would find that an pineapple gene or a chromosomal region would have two best matched or orthologous rice genes or chromosomal regions, and two secondary or out paralogous genes or chromosomal regions. In that there have often been gene or DNA losses after polyploidization(s), the above 1:2 ratio may not hold for all collinear genes revealed above. Here, we managed to classify the intergenomic blocks by considering the gene and chromosomal segmental similarities, and complement DNA breakages to distinguish orthologous from out paralogous blocks. Gene similarity was measured by inferring synonymous nucleotide substitutions (*K*s). Chromosomal segmental similarity was measured by using *K*s median of genes in the collinear blocks, and collinear gene numbers. Often without much difficulty, we managed to distinguish the orthologs from out paralogous blocks (**Figure 3**). The *K*s corresponding to the split of two plants are often 0.95–1.20; while those around 1.5 may be related to homologs produced by more ancient events. For example, pineapple chromosomes 1, 20, and 21 (Ac01, Ac20, and Ac21), have orthologous regions in Os02 and Os04; and Ac05, Ac06, and Ac19 have orthologous regions in Os02 and Os04 (**Figure 3**). Sometimes, based on *K*s median, it was difficult to distinguish orthologous and out paralogous regions. At these few cases, we had to make a close check of the collinear genes shared by these blocks. For example, one end of Ac19 has prominent homology with Os02, Os04, and Os06 (**Figure 3**), and the corresponding *K*s medians are also similar. A close check of the collinear genes showed that Ac19 had 107, 51, and 135 collinear genes with the three rice chromosomes, respectively, indicating that Os02 and Os06 are much more similar to Ac19 than Os04. As to the collinear gene content, we found Os02 shared 45 or 42.05% of their collinear genes with Os06, but only 5 or 4.7% of their collinear genes with Os04. This finding showed clearly that Os02 and Os06 are orthologous copies of Ac19, while Os04 is out paralogous one. Eventually, we divided the orthologous copies from the out paralogous ones in the whole comparison of two genomes (**Supplementary Table 1**).

### Inference of Chromosome Fusions

**FIGURE 4:**
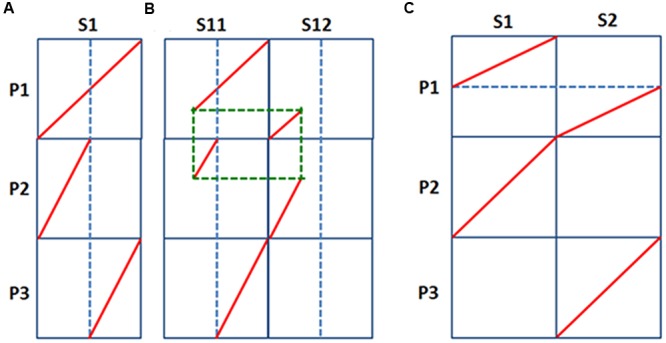
**Fission and fusion models of chromosome rearrangements.** A comparison of chromosomes from species S, and their duplicated copies in another species P. **(A)** A chromosome duplicate broke, and each split segment were merged to produce two independent chromosomes; **(B)** Besides the conditions in A, a chromosome fission occurred in species S; **(C)** Two independent chromosomes existed in P, and after whole-genome doubling, one set of chromosome fused to produce a merged chromosome.

To solve the standing controversy of basic chromosome number in ancestral grass genome before the grass-common tetraploidization, we considered the corresponding orthologous segments between pineapple and rice. Let us first illustrate the two models: fission model and fusion model understand the circumstance of a grass-common tetraploidization (**Figure 4**).

For the fission model, considering that there was an chromosome S1, preserved in pineapple, it should have corresponded to P1 in the grass ancestor before the tetraploidy, and then duplicated to two after tetraploidization to get two chromosomes P1, one of which split into two to produce P2 and P3 (**Figure 4A**). That is, chromosome S1 may correspond to a full rice chromosome P1, and at the meantime, each of them are the direct addition of rice chromosomes P2 and P3. The content of S1 chromosome may exist in segments due to genomic rearrangements, therefore may exist in two or more extant chromosomes, S11 and S12, which may share common breakage points, linking them to make a complete S1 (**Figure 4B**). Considering possibility of more ancient polyploidization, there might have more ancient duplicates in pineapple genome.

For the fusion model, considering that there were two ancestral chromosomes preserved in pineapple, S1 and S2, they should have corresponded to P2 and P3 in grass common ancestor, which were duplicated to get two P2 and two P3, and later one P2 and one P3 merged to produce another grass chromosome P1 (**Figure 4C**). The S1 and S2 chromosomes might be affected by genomic rearrangements to have it gene content separated to exist in extant pineapple chromosomes. Alternatively, they could have more ancient duplicates in pineapple. Nonetheless, if the fusion model is right, for S1 and S2 were independent chromosomes, we anticipate that they correspond to different sets of grass chromosomes.

**FIGURE 5:**
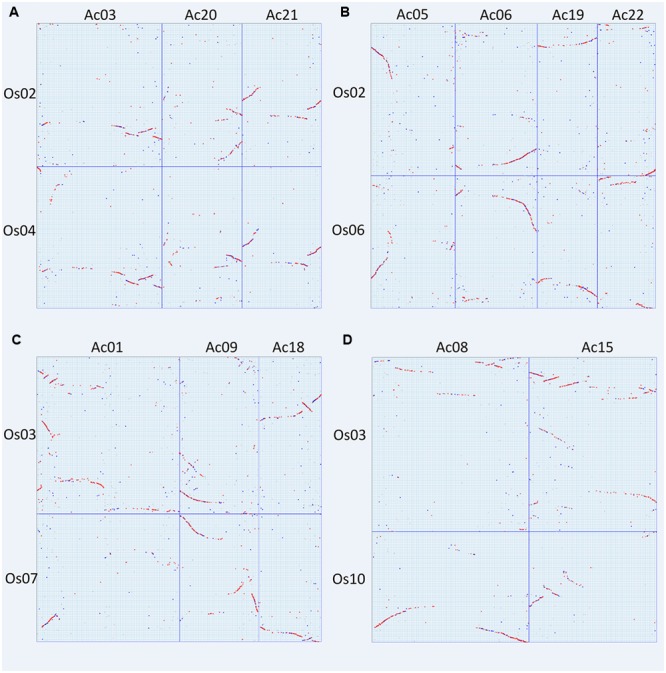
**Specific chromosome dotplots between specific chromosomes. (A)** Rice chromosomes 2 and 4 to pineapple chromosomes 3, 20, and 21; **(B)** rice chromosomes 2 and 6 to pineapple chromosomes 5, 6, 19 and 22; **(C)** rice chromosomes 3 and 7 to pineapple chromosomes 1, 9, and 18; **(D)** rice chromosomes 3 and 10 to pineapple chromosomes 8 and 15.

As to the grass chromosomes Os02, Os04, and Os06, we found that they followed a fusion model. Os02 has orthologous correspondence in Ac03, Ac05, Ac06, Ac19, Ac20, Ac21, and Ac22, showing that it was composed of DNA segments from different pineapple chromosomes. Os04 has orthologous correspondence in Ac03, Ac20, and Ac21 (**Figure 5A**); while Os06 has orthologous correspondence with Ac05, Ac06, Ac19, and Ac22 (**Figure 5B**). This shows a perfect independent correspondence of Os04 and Os06 in pineapple genome, strongly supporting the fusion model.

As to the grass chromosomes Os03, Os07, and Os10, we found that they also follow a fusion model. Os03 has orthologous correspondence in Ac01, Ac07, Ac08, Ac09, Ac15, and Ac18. Os07 has orthologous correspondence in Ac01, Ac09, and Ac18 (**Figure 5C**); while Os10 has orthologous correspondence with Ac08 and Ac15 (**Figure 5D**). This shows a perfect independent correspondence of Os07 and Os10 in pineapple genome, also strongly supporting the fusion model.

**FIGURE 6:**
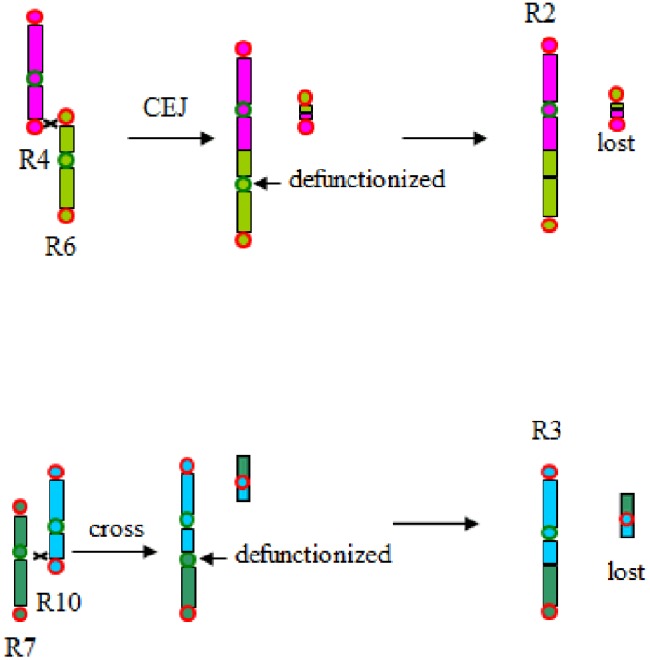
**Chromosome fusion and evolutionary changes.** CEJ, chromosome end–end joining; CFI, chromosome fission (shown by crosses). Chromosomes were colored as to a scheme adopted in Wang et al. (2015b). Rice chromosomes 4 and 6 cross-over near their telomeres, then lost a tiny part to produce chromosome 2. Rice chromosomes 7 and 10 cross-over near their telomeres, then lost a tiny part to produce chromosome 3.

Therefore, together with the previous analysis published (Wang et al., 2016), the present analysis clearly shows that the grass common ancestor had seven basic chromosomes before the grass-common tetraploidization. This shows that chromosome number reduction often occurred after polyploidization, supporting our previous proposition of a telomere-centered genomic repatterning process (**Figure 6**).

### Genomic Fractionation in Rice

Wide-spread gene losses occurred in rice after the split with pineapple. As to inferred gene collinearity, we found that 18101 or 75.76% of pineapple genes could not find their collinear orthologs at expected locations in rice, only a tiny fraction of 4.59% have both duplicate copies preserved, and 19.65% have only one alternative copy preserved in rice, showing a large-scale fractionation in rice and possibly other grasses. This may possibly be resulted from gene losses after the grass-common tetraploidization. However, we found in these 18101 genes, 69.6% of them have BLASTP hits at 1*e*-10 or smaller, about two thirds are bidirectional best hits, and only ∼5500 genes did not have hits in rice. These facts showed the likelihood of the existence of their rice orthologs, which might have been transposed to other chromosomal locations after their split, and also showed that only ∼1/5 (∼5500/18101) of the pineapple genes might be totally lost in rice and other grasses.

## Discussion

Rice is among the most researched model plants for its economic importance to human food nutrient (International Rice Genome Sequencing Project, 2005; The-3000-rice-project, 2014). More and more evidences show that it is a good model plant to understand grass origination and evolution (Wang et al., 2015b). On one hand, it has been the slowest evolving plant, as compared to other grasses, at least 15% slower than maize, sorghum, and other grasses sequenced so far (Wang et al., 2015b). On the other hand, it has a genome structure most resembling that of grass ancestors. Comparative genomics analysis of rice and other grasses reported a 5 or *n* = 7 basic chromosomes before the grass-common tetraploidization (Salse et al., 2009; Wang et al., 2015a). Recently, an effort to distinguish two subgenomes, dominant and sensitive ones, let the authors favor the hypothesis of *n* = 7 proto-chromosomes (Murat et al., 2015). That is, a *n* = 7 proto-chromosome model could help define and separate dominant subgenome from sensitive one, whereas a *n* = 5 proto-chromosome model cannot make it. Though the discussion is reasonable, this is seemingly like to prove a new hypothesis, and have to negate an old hypothesis. Therefore, an independent analysis is necessary to solve the controversy and the availability of the pineapple provided such a precious opportunity. The good thing is that pineapple was not affected by polyploidization(s) after its split from grasses, making it have a relatively simple genome structure, and be a valuable reference to understand those of grasses.

Here, let us discuss a little about the genome stability of an allopolyploid, which would shed light on the genome structure of grasses and the nature of the grass-common tetraploidization. Recently, a neo-tetraploid, *Brassica napus* (AACC) was sequenced, and it was inferred to form only ∼7500 years ago, with parental lines of *B. rapa* (AA) and *B. oleracea* (CC; Chalhoub et al., 2014). Amazingly, very few genes (<200) might have been deleted after the formation of the tetraploid. While for the grass-common tetraploid ancestor, it was reported that there should have been massive gene losses in that only ∼30% genes in collinearity likely produced by the tetraploidization were preserved in the extant genomes (Paterson et al., 2004; Wang et al., 2005). This was inferred by alternative gene missing in the duplicated regions.

Actually, the alternative missing genes can be resulted from the following scenarios: (1) gene losses or translocations in the parental lines before their hybridization; (2) gene losses or translocations during the early days after tetraploidization; (3) gene losses or translocations during the following time much after tetraploidization. The first scenario describes just like that of the Brassica plants. The *B. rapa* and *B. oleracea* have genomes with prominent difference in gene numbers. The hybridization of these divergent parents would produce an amphibian or allopolyploid with quite stable genome, for illegitimate recombination may be much restricted. Though illegitimate recombination may still occur to lead to gene conversion, as observed in *B. napus*, it may not result in massive gene losses. The third scenario may be possible but slim in chance, for rice and sorghum a comparison showed that they each preserved >97% of their gene collinear genes after their split, showing a very stable genome after millions of years of the tetraploid formation (Paterson et al., 2009). The second scenario shows that the parental lines were not very divergent and a lot illegitimate recombination will occur, resulting in massive gene losses and chromosomal rearrangement. This may not explain what we observed in rice, in that, most of the ancestral genome structure after the tetraploidization have been preserved in rice for tens of millions of years, only with 14 chromosomes rearranged to reach 12 chromosomes after two chromosome fusions, as described above. Therefore, we favor the first possibility that two prominently divergent genomes merged to produce the grass tetraploid ancestor, which was an amphibian tetraploid or allotetraploid with a considerably stable genome.

Here, we provided a solid evidence for the *n* = 7 or 2n = 2x = 14 proto-chromosome model, and solved the controversy. As to above discussion, the grass-common tetraploidization is in essence or mostly an amphibian one, resulting in an allotetraploidization. This conclusion is consistent with previous finding by considering of genomic plasticity (Murat et al., 2014). Therefore, after the tetraploidization the grass ancestor should have followed diploid hybridity, and it should have 2n = 4x = 28 chromosomes. Then, these chromosomes were subjected to a few fusion events, to have 2n = 24 chromosomes in a common ancestor the extant sequenced grasses, as still preserved in rice, while further reduced in many other grasses.

## Author Contributions

The study was conceived by XW and JW. YP, JiaY, PS, RX, JigY, YuL, YiL, XM, NY, TL, and XL contributed to data collection and bioinformatics analysis. JW, XW, YP, and JiaY participated in preparing and writing the manuscript. ZW, LW, WG, XS, SS, TL, and DJ performed the analysis with constructive discussions. All authors contributed to revising the manuscript. All authors had read and approved the final manuscript.

## Conflict of Interest Statement

The authors declare that the research was conducted in the absence of any commercial or financial relationships that could be construed as a potential conflict of interest.

## Footnotes

1 https://phytozome.jgi.doe.gov/pz/portal.html

## Supplementary Material

TABLE S1 **Homologous alignments of rice and pineapple**.

## References

[B1] Chalhoub B. Denoeud F. Liu S. Parkin I. A. Tang H. Wang X. (2014). Plant genetics. Early allopolyploid evolution in the post-neolithic *Brassica napus* oilseed genome. *Science* 345 950–953. 10.1126/science.1253435 25146293

[B2] Ding D. Q. Yamamoto A. Haraguchi T. Hiraoka Y. (2004). Dynamics of homologous chromosome pairing during meiotic prophase in fission yeast. *Dev. Cell* 6 329–341. 10.1016/S1534-5807(04)00059-0 15030757

[B3] Grass-phylogeny-working-group-II (2012). New grass phylogeny resolves deep evolutionary relationships and discovers C4 origins. *New phytol.* 193 304–312. 10.1111/j.1469-8137.2011.03972.x 22115274

[B4] International Rice Genome Sequencing Project (2005). The map-based sequence of the rice genome. *Nature* 436 793–800. 10.1038/nature03895 16100779

[B5] Jordan P. (2006). Initiation of homologous chromosome pairing during meiosis. *Biochem. Soc. Trans.* 34(Pt 4) 545–549. 10.1042/BST0340545 16856856

[B6] McKee B. D. (2004). Homologous pairing and chromosome dynamics in meiosis and mitosis. *Biochim. Biophys. Acta* 1677 165–180. 10.1016/j.bbaexp.2003.11.017 15020057

[B7] Murat F. Xu J. H. Tannier E. Abrouk M. Guilhot N. Pont C. (2010). Ancestral grass karyotype reconstruction unravels new mechanisms of genome shuﬄing as a source of plant evolution. *Genome Res.* 20 1545–1557. 10.1101/gr.109744.110 20876790 PMC2963818

[B8] Murat F. Zhang R. Guizard S. Flores R. Armero A. Pont C. (2014). Shared subgenome dominance following polyploidization explains grass genome evolutionary plasticity from a seven protochromosome ancestor with 16K protogenes. *Genome Biol. Evol.* 6 12–33. 10.1093/gbe/evt200 24317974 PMC3914691

[B9] Murat F. Zhang R. Guizard S. Gavranovic H. Flores R. Steinbach D. (2015). Karyotype and gene order evolution from reconstructed extinct ancestors highlight contrasts in genome plasticity of modern rosid crops. *Genome Biol. Evol.* 7 735–749. 10.1093/gbe/evv014 25637221 PMC5322550

[B10] Nicolas S. D. Leflon M. Liu Z. Eber F. Chelysheva L. Coriton O. (2008). Chromosome ‘speed dating’ during meiosis of polyploid *Brassica* hybrids and haploids. *Cytogenet. Genome Res.* 120 331–338. 10.1159/000121082 18504362

[B11] Paterson A. H. Bowers J. E. Bruggmann R. Dubchak I. Grimwood J. Gundlach H. (2009). The *Sorghum* bicolor genome and the diversification of grasses. *Nature* 457 551–556. 10.1038/nature07723 19189423

[B12] Paterson A. H. Bowers J. E. Chapman B. A. (2004). Ancient polyploidization predating divergence of the cereals, and its consequences for comparative genomics. *Proc. Natl. Acad. Sci. U.S.A.* 101 9903–9908. 10.1073/pnas.0307901101 15161969 PMC470771

[B13] Salse J. Abrouk M. Murat F. Quraishi U. M. Feuillet C. (2009). Improved criteria and comparative genomics tool provide new insights into grass paleogenomics. *Brief. Bioinform.* 10 619–630. 10.1093/bib/bbp037 19720678

[B14] The-3000-rice-project (2014). The 3,000 rice genomes project. *GigaScience* 3:7 10.1186/2047-217X-3-7 PMC4035669 24872877

[B15] Vazquez J. Belmont A. S. Sedat J. W. (2002). The dynamics of homologous chromosome pairing during male *Drosophila* meiosis. *Curr. Biol.* 12 1473–1483. 10.1016/S0960-9822(02)01090-4 12225662

[B16] Wang X. Jin D. Wang Z. Guo H. Zhang L. Wang L. (2015a). Telomere-centric genome repatterning determines recurring chromosome number reductions during the evolution of eukaryotes. *New Phytol.* 205 378–389. 10.1111/nph.12985 25138576

[B17] Wang X. Shi X. Hao B. Ge S. Luo J. (2005). Duplication and DNA segmental loss in the rice genome: implications for diploidization. *New Phytol.* 165 937–946. 10.1111/j.1469-8137.2004.01293.x 15720704

[B18] Wang X. Shi X. Li Z. Zhu Q. Kong L. Tang W. (2006). Statistical inference of chromosomal homology based on gene colinearity and applications to *Arabidopsis* and rice. *BMC Bioinformatics* 7:447 10.1186/1471-2105-7-447 PMC1626491 17038171

[B19] Wang X. Wang J. Jin D. Guo H. Lee T. H. Liu T. (2015b). Genome alignment spanning major Poaceae lineages reveals heterogeneous evolutionary rates and alters inferred dates for key evolutionary events. *Mol. plant* 8 885–898. 10.1016/j.molp.2015.04.004 25896453

[B20] Wang X. Wang Z. Guo H. Zhang L. Wang L. Li J. (2016). Telomere-centric genome repatterning determines recurring chromosome number reductions during the evolution of eukaryotes. *New phytol.* 205 378–389. 10.1111/nph.12985 25138576

[B21] Yang Z. (2007). PAML 4: phylogenetic analysis by maximum likelihood. *Mol. Biol. Evol.* 24 1586–1591. 10.1093/molbev/msm088 17483113

